# Effect of high-flow nasal cannula on patients’ recovery after inhalation general anesthesia

**DOI:** 10.12669/pjms.39.3.6638

**Published:** 2023

**Authors:** Dong Liu, Teng-yu Jin, Wei Li, Li Chen, Dong Xing

**Affiliations:** 1 Dong Liu, Department of Anesthesiology, Baoding No.1 Hospital, Baoding 071000, Hebei, China; 2 Teng-yu Jin, Department of Clinical Medicine, School of Basic Medicine, Hebei Medical University, Shijiazhuang 050017, Hebei, P.R.China; 3 Wei Li, Department of Anesthesiology, Longyao county hospital, Xingtai 055350, Hebei, China; 4 Li Chen, Department of General Medicine, The Fourth Hospital of Hebei Medical University, No.12 of Jiankang Road, Chang’an District, Shijiazhuang 050011, Hebei, China; 5 Dong Xing Department of Emergency, The Fourth Hospital of Hebei Medical University, No.12 of Jiankang Road, Chang’an District, Shijiazhuang 050011, Hebei, China

**Keywords:** High-flow nasal cannula, Inhalation, General anesthesia, Recovery period, Effect

## Abstract

**Objective:**

To investigate the effect of high-flow nasal cannula (HFNC) and Oxygen Nebuliser mask (ONM) on patients recovering from inhalation anesthesia.

**Methods:**

A retrospective analysis was performed on 128 patients after inhalation of general anesthesia in the recovery room of the Anesthesiology Department of The Fourth Hospital of Hebei Medical University from September 2019 to September 2021. All patients received the same anesthesia induction and analgesia methods, inhalation anesthesia or intravenous-inhalation anesthesia maintenance, recovered spontaneous breathing and removed endotracheal intubation after surgery, then were divided into HFNC group and ONM group for oxygen therapy. HFNC setting mode: flow rate: 20-60 L/minutes, humidification temperature: 37°C, the oxygen concentration was adjusted to maintain finger pulse oxygen saturation SPO_2_>90%; ONM group, the oxygen flow rate was adjusted to maintain finger pulse oxygen saturation SPO_2_>90%. All patients in the two groups were compared immediately after they entered the recovery room for 0 minutes,, 10 minutes, and 20 minutes,, including tidal volume, blood gas, Richmond Agitation-Sedation Scale (RASS) score and time from sedation to awakening.

**Results:**

The changes in tidal volume, oxygenation index and RASS score over time in the HFNC group were higher than those in the ONM group (*p<*0.05), and the awakening time in the HFNC group was faster than that in the ONM group (*p<*0.01), with significant statistical differences.

**Conclusions:**

Compared with ONM, HFNC can shorten postoperative recovery time, reduce the incidence of agitation and improve lung function and oxygenation state during recovery from anesthesia.

## INTRODUCTION

Delayed awakening is common in patients with inhalation anesthesia or intravenous inhalation combined anesthesia due to incomplete drug metabolism. Patients with the delayed awakening are frequently accompanied by postoperative agitation.^1^,^2^ Delayed awakening and agitation generally lead to the change of abnormal physiological indexes. Patients are physiologically showing nausea and vomiting, increased blood pressure, faster heart rate and increased myocardial oxygen consumption. However, in the absence of timely control and treatment, it may possibly increase the risk of accidental injury and serious cardiovascular complications.^3^ Agitation during recovery from anesthesia has an intimate association with the metabolism of anesthetics, especially the incomplete clearance of inhaled anesthetics *in vivo*. The clearance of inhaled anesthetics depends primarily on adequate pulmonary ventilation, and ensuring effective alveolar ventilation has been recognized to be an important prerequisite for awakening. It is critical to supply patients with appropriate oxygen therapy mode during perioperative anesthesia to ensure adequate pulmonary ventilation and oxygen supply, so as to avoid postoperative recovery delay and agitation as much as possible.^4^ Accordingly, a retrospective cohort analysis was performed in this study to investigate the effect of high-flow nasal cannula (HFNC) and Oxygen Nebuliser mask (ONM) on patients recovering from inhalation anesthesia.

## METHODS

The subjects of the study were 128 patients after general anesthesia in the recovery room of the Department of Anesthesiology in The Fourth Hospital of Hebei Medical University from September 2019 to September 2021. All patients were provided with the same anesthesia induction method: prior to induction, intravenous injection of 0.5~1 ug/kg remifentanil (Yichang Humanwell Pharmaceutical Co., Ltd.) for over one minutes,, 0.3~0.6 mg/kg cis-Atracurium Besylate (Jiangsu Hengrui Medicine Co., Ltd.), Propofol Medium and Long Chain Fat Emulsion Injection (1.5~2.5 mg/kg). Intraoperative maintenance: Continuous inhalation of isoflurane or sevoflurane, and continuous pumping of remifentanil. The depth of anesthesia and invasive arterial blood pressure were monitored. Mechanical ventilation was performed in volume control mode. After the operation, the endotracheal intubation was removed when the patients had the Richmond Agitation-Sedation Score (RASS)= -3 points,^5^ recovered spontaneous breathing, with a tidal volume of >5 ml/kg and stable vital signs. Then, the patients were sent to the anesthesia recovery room for oxygen inhalation with ONM, with the adjustment of the oxygen flow rate and maintenance of the finger pulse oxygen saturation SPO_2_≥90%. The study was approved by the Institutional Ethics Committee of The Fourth Hospital of Hebei Medical University (Ethics No.: 2020ky054) at April 18, 2022

### Inclusion criteria:

- Patients aged between 18 and 75 years old;
- Patients with the following mode of anesthesia maintenance: endotracheal intubation+intravenous and/or inhalation anesthesia;
- Patients with the body mass index (BMI) ranging between 18<BMI≤25
- Patients who agreed to analgesic treatment with the analgesic pump after operation.

### Exclusion criteria:

- Patients with anesthesia time<2 h or >5 h;
- Patients whose stay time in the anesthesia recovery room was less than observation time;
- Patients with a history of drug abuse and psychotropic drug dependence
- Patients with interstitial pneumonia or chronic obstructive pulmonary disease (COPD) grade II (FEV_1_≤50%) or above;
- Patients undergoing lobectomy in thoracic surgery.

### Observational indexes:

The incidence of agitation during recovery room in the two groups, in which patients with RASS score >3 points were diagnosed as agitation.^5^ The time to reach the state of fully awake and autonomous eye opening in the two groups, i.e., RASS score ranging from -3 to 0. The change trend of tidal volume and arterial oxygenation index at 0 min (immediately after entering the recovery room), 10 minutes,and 20 minutes, and when the patient was fully awake (RASS score=0). The change trend of RASS score at different time points (0 minutes, 10 minutes, and 20 min) between the two groups.

### Statistical analysis:

SPSS software 22.0 was used for statistical analysis and processing. The measurement data were expressed in x±s. The measurement data were examined by t-test and the counting data were tested by χ^2^ test. P*<*0.05 indicated the existence of a statistical difference and *p<*0.01 suggested the presence of a significant statistical difference.

## RESULTS

A total of 128 patients were included in this study, including 63 in the HFNC group and 65 in the ONM group. There were statistical differences in partial pressure of carbon dioxide (PCO_2_), the number of patients with COPD, the incidence of agitation and the awakening time (RASS=0 point) between the two groups (*p<*0.05) (Table-I).

**Table-I:** Comparison of general data between groups(n1+n2 =128)

	HFNC (n=63)	ONM (n=65)	χ2/t value	p value
***Gender [n(% )]***			0.035	0.851
Female	29 (46.0)	31 (47.7)		
Male	34 (54.0)	34 (52.3)		
Age (years,*χ̅*±*S* )	61.8±12.3	62.6±10.7	-0.403	0.688
BMI	21.7±2.7	22.4±2.4	-1.510	0.134
***Inhaled drug [n(%)]***			
Isoflurane	30 (47.6)	37 (57.0)	1.110	0.292
Sevoflurane	33 (52.4)	28 (43.0)		
***Surgical site [n(%)]***				
Gynaecology	22 (35.0)	20 (30.8)	0.250	0.617
Gastrointestinal surgery	19 (30.1)	21 (32.3)	0.069	0.793
Neurosurgery	10 (15.9)	9 (13.8)	0.104	0.747
Orthopaedics	12 (19.0)	15 (23.1)	0.312	0.576
Surgical time (hours, *χ̅*±*S* )	3.2±0.6	3.1±0.5	0.593	0.554
***Physiological index***				
Mean arterial pressure (mmHg, *χ̅*±*S* )	82. 6±8.6	84.0±8.3	-0.882	0.380
Heart rate (times/min, *χ̅*±*S*)	73.5±12.2	76. 3±11.8	-1.279	0.203
Respiratory rate (times/min, *χ̅*±*S*)	21.8±3.9	22.7±4.1	-1.278	0.203
Tidal volume (ml/kg, *χ̅*±*S*)	6.5±0.9	6.3±0.7	1.596	0.113
***Blood gas index***	39. 6±2.2	38.3±2.5	2.876	0.005
Partial pressure of carbon dioxide (PCO2) (mmHg, *χ̅*±*S* )	377.5±23.2	379.7±14.6	-0.649	0.517
Oxygenation index (PO2/FiO2)				
***Underlying disease [n(%)]***				
Type II diabetes mellitus	8 (12.7)	11 (16.9)	0.452	0.501
Chronic obstructive pulmonary disease	11 (14.3)	4 (7.7)	3.953	0.047
Coronary heart disease (NYHA cardiac function grade I ~ II)	11 (17.5)	14 (21.5)	0.339	0.561
VAS score of awake state (*χ̅*±*S*)	5.2±1.6	5.5±1.4	-1.410	0.161
***Incidence rate of restlessness [n(% )]***				
Inhaling isoflurane	3 (10)	9 (30)	3.764	0.052
Inhaling sevoflurane	3 (10)	7 (10.8)	2.797	0.094
Total	6 (9.5)	16 (24.6)	5.119	0.023
Awakening (RASS=0 point) time (min, *χ̅*±*S*)	27.9±4.4	32.6±5.3	-5.331	0.000

**Fig.1:**
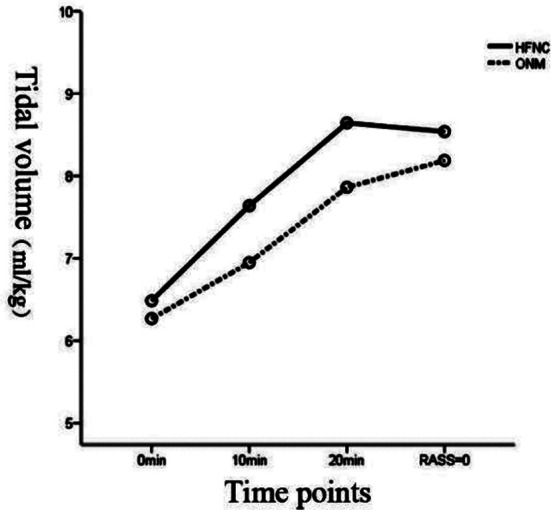
Tidal volume profile.

As shown in Table-II, the tidal volume was higher in the HFNC group than that in the ONM group after entering the recovery room for 10 minutes, and 20 minutes, to awakening (RASS=0 point), with a statistical difference (*p<*0.05). The corresponding observation time interacted with the oxygen inhalation mode (F=4.829, *p*=0.005<0.01). Besides, there was a difference in tidal volume between the two different oxygen inhalation modes (F=46.119, *p*=0.000<0.01, Fig.1).

**Table-II:** Comparison of tidal volume between the two groups at different times (*χ̅*±*S*) n1+n2 =128, ml/kg,*χ̅*±*S*.

Groups	0 min	10 min	20 min	Awakening (RASS=0 point)	F value	p value
HFNC group	6.5±0.9	7.6±0.7^*Δ^	8.6±0.5^*Δ^	8.5±1.2^#^	150.327	0.000
ONM group	6.3±0.7	7.0±0.5^*Δ^	7.9±0.6^*Δ^	8.2±0.6^#^	109.594	0.000
F value	2.547	42.222	64.061	4.360		
p value	0.113	0.000	0.000	0.000		

***Note:*** Compared with the value at 0min, ^*^p<0.01; Compared with the previous time point, ^Δ^p<0.01,

# p<0.05.

**Fig.2:**
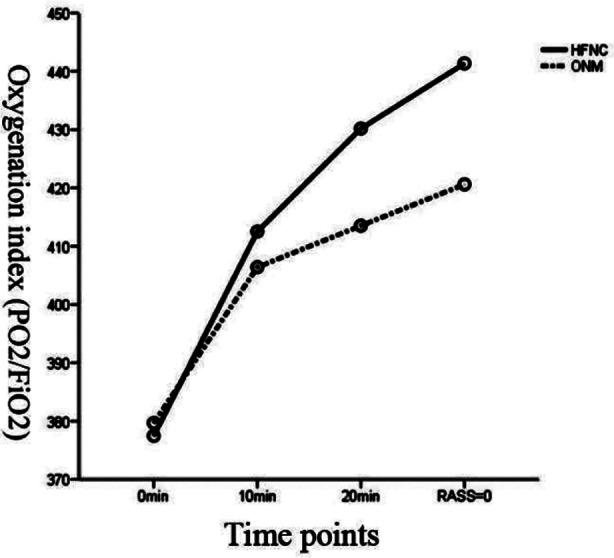
Oxygenation index (PO_2_/FiO_2_) profile.

As shown in Table-II, there was a statistical difference that PO_2_/FiO_2_ was higher in the HFNC group than that in the ONM group after entering the recovery room for 10 minutes, and 20 minutes, to awakening (RASS=0 point) (*p<*0.05). The corresponding observation time interacted with the oxygen inhalation mode (F=16.763, *p*=0.000<0.01), and a difference was observed in PO_2_/FiO_2_ between the two different oxygen inhalation modes (F=30.250, *p*=0.000<0.01, Fig.2).

**Fig.3:**
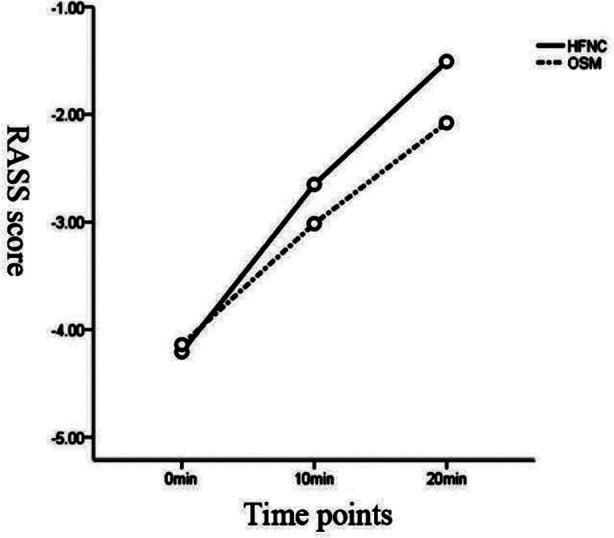
RASS score profile.

As shown in Table-IV, there was a statistical difference that the RASS scores was higher in the HFNC group than that in the ONM group after entering the recovery room for 10 minutes, and 20 minutes, (*p<*0.05). The corresponding observation time interacted with the oxygen inhalation mode (F=7.044, *P*=0.002<0.01), showing a difference in RASS score between the two different oxygen inhalation modes (F=10.861, *P*=0.000<0.01, Fig.3).

**Table-III:** Comparison of oxygenation index (PO_2_/FiO_2_) between the two groups at different times - n1+n2 =128,*χ̅*±*S*.

Groups	0 min	10 min	20 min	Awakening (RASS=0 point)	F value	p value
HFNC group	377.5±23.2	412.5±20.0^*^	430.2±14.5^*Δ^	441.3±15.7^*Δ^	163.711	0.000
ONM group	379.7±14.6	406.4±14.9^*Δ^	413.5±13.1^*Δ^	420.6±12.0^*#^		
F value	0.421	3.850	46.454	70.600	71.492	0.000
p value	0.517	0.052	0.000	0.000		

***Note:*** Compared with the value at 0min, ^*^p<0.01; Compared with the previous time point, ^Δ^p<0.01, ^#^p<0.05.

**Table-IV:** Comparison of RASS scores between the two groups at different times (*χ̅*±*S*), n1+n2 =128, *χ̅*±*S*.

Groups	0 min	10 min	20 min	F value	p value
HFNC group	-4.2±0.8	-2.7±0.8^*Δ^	-1.5±0.8^*Δ^	227.612^*Δ^	0.000
ONM group	-4.1±0.8	-3.0±0.7^*Δ^	-2.1±0.6^*Δ^	139.928^*Δ^	0.000
F value	0.238	7.116	21.620		
p value	0.627	0.000	0.000		

***Note:*** Compared with the value at 0min, ^*^p<0.01; Compared with the previous time point, ^Δ^p<0.01.

**Fig.4:**
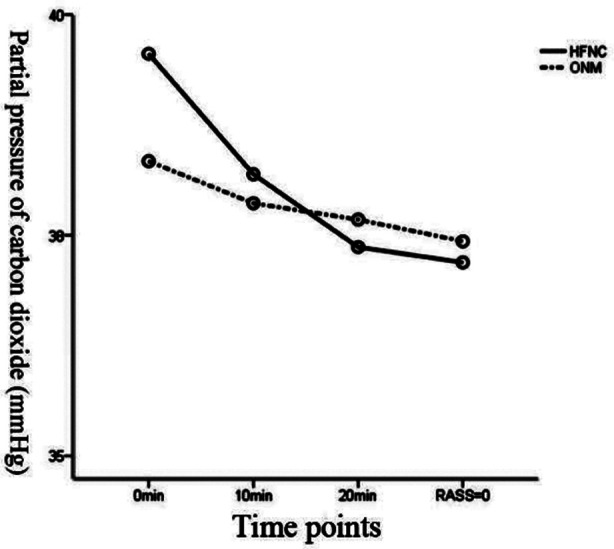
Partial pressure of carbon dioxide profile.

There was a statistical difference in PCO_2_ between the two groups immediately after entering the recovery room (0 min) (*p<*0.05). A decreased trend of PCO_2_ was observed in the HFNC group after entering the recovery room for 10 min and 20 min to awakening (RASS=0 point) (*p<*0.05), as shown in Table-V. The corresponding observation time interacted with the oxygen inhalation mode (F=5.452, *p*=0.002<0.01), yet without difference in PCO_2_ under different oxygen inhalation modes (F=1.788, *p*=0.184>0.05, Fig.4).

**Table-V:** Comparison of partial pressure of carbon dioxide (PCO_2_) between the two groups (*χ̅*±*S*) n1+n2 =128, mmHg,*χ̅*±*S*.

Groups	0 min	10 min	20 min	Awakening (RASS=0 point)	F value	p value
HFNC group	39.6±2.3	38.2±1.6^*Δ^	37.4±1.5^*#^	37.2±1.6^*#^	15.618	0.000
ONM group	38.3±2.5	37.9±1.6	37.7±1.5	37.4±1.7		
F value	8.269	1.409	1.339	0.685	2.095	0.104
p value	0.005	0.237	0.249	0.409		

***Note:*** Compared with the value at 0min, ^*^p<0.01; Compared with the previous time point, ^Δ^p<0.01, ^#^p<0.05.

## DISCUSSION

Delayed awakening is defined medically as a state of unresponsiveness or deep sedation from which the patient cannot be aroused 30-60 minutes after the end of the operation and cannot restore an adequate level of consciousness.^6^ In addition to increasing the burden on patients, the emergence of some complications are also quite difficult for anesthesiologists to deal with.^7^ Inappropriate intraoperative ventilation management techniques may involve hyperventilation/hypoventilation. It may result in the prolongation of the time from sedation to awakening owing to the presence of hypocapnia and cerebral vasoconstriction (hyperventilation) or CO_2_ anesthesia (hypoventilation).^8^ Ensuring adequate alveolar ventilation during awakening to eliminate inhalational anesthetics *in vivo* in a timely manner is the premise of rapid recovery of consciousness. In addition to providing high-concentration oxygen inhalation, HFNC can output constant oxygen concentration at a constant temperature of about 37°C and relative humidity of 100%.^9^,^10^ Moreover, HFNC can also produce positive airway pressure in the exhalation airway, effectively improving the oxygenation state.^11^,^12^

In this study, the HFNC group had a higher proportion of patients with COPD and a relatively high PCO_2_. However, with the extension of treatment time, PCO_2_ decreased significantly in patients from this group. It may be explained by the fact that FNC produces certain PEEP in the respiratory tract of patients, offsetting part of endogenous PEEP. Simultaneously, although the HFNC group had a higher proportion of COPD patients and PCO_2_, there was no significant difference in PCO_2_ between the two groups at 10 minutes, and 20 minutes,with the progress of treatment time. The results suggest that HFNC may improve the CO_2_ retention status of COPD patients to some extent.^13^

Due to prolonged operation and high-concentration oxygen inhalation during anesthesia, patients may have partial lung collapse frequently after anesthesia.^14^,^15^ In case of a failure to correct in time, it may lead to the unbalanced ratio of ventilation and blood flow due to the reduction of lung volume, and then hypoxemia, resulting in delayed awakening. ONM is a routine therapeutic strategy in the perioperative period, especially after the operation. Nevertheless, it has a disadvantage that it can not provide reliable FiO_2_ or reduce patients’ work of breathing.^16^,^17^ HFNC can make up for its limitations. As for the possible reason, HFNC can increase mucus clearance rate, and improve dead space volume and lung mechanics, thereby improving the tidal volume and ensuring effective alveolar ventilation. In our study, after entering the recovery room for 10 min and 20 min, the tidal volume of patients in the HFNC group increased rapidly compared with that in the ONM group, which was similar to the results of previous studies.^18^ Similarly, the tidal volume in the HFNC group was significantly higher than that in the ONM group at all time points, also suggesting that HFNC can improve the tidal volume of patients with routine postoperative extubation.

Oxygenation index reflects the oxygenation status of patients. In addition to maintaining a certain oxygen concentration, improving the diffusion function of alveoli is also effective to ensure the oxygenation state of patients. In this study, HFNC could significantly improve PO_2_/FiO_2_ in postoperative extubation patients. The oxygenation index (PO_2_/FiO_2_) profile in Fig.2 shows that there was a difference in PaO_2_/FiO_2_ under the two different oxygen inhalation modes. Which was similar to the results of previous studies.^19^

The awakening time of patients depends largely on the clearance speed of anesthetics *in vivo*. All patients in our study were provided with cis-Atracurium Besylate with strong effect, no accumulation, rapid metabolism and rapid recovery.^20^ Findings in our study revealed that the awakening time was significantly shortened in the HFNC group than that in the ONM group. Simultaneously, the score was higher in the HFNC group than that in the ONM group ten minutes after entering the recovery room. Besides, the RASS score profile indicated the existence of a difference in the score between the two groups under the two different oxygen inhalation modes.

These data suggest that compared with ONM, HFNC can accelerate the clearance of inhalational anesthetics in the lung and shorten the awakening time of patients under inhalation general anesthesia. In addition, the incidence of agitation in the HFNC group was lower than that in the ONM group. It may be related to the rapid clearance of inhalational anesthetics in the HFNC group, leading to a relatively faster awakening of patients.

### Limitation of this study:

Firstly, the flow rate of HFNC was set within the range of 20-60 L/ minutes,according to the patients’ own condition and the independent choice of doctors and nurses. Therefore, it remains to be further discussed concerning the specific flow rate value to produce effective PEEP in future research. Secondly, our study did not strictly distinguish between inhalation anesthesia and combined intravenous and inhalation anesthesia, without the consideration of the effect of intravenous anesthetics consequently.

## CONCULSION

To sum up, findings in our study suggest that compared with ONM, HFNC can shorten postoperative recovery time, reduce the incidence of agitation and improve lung function and oxygenation state during recovery from anesthesia.

### Authors’ Contributions:

**DL and DX** designed this study, prepared this manuscript, are responsible and accountable for the accuracy and integrity of the work.

**TJ and WL** collected and analyzed clinical data.

**LC:** Data analysis**,** significantly revised this manuscript.
